# Application of Long-Acting VLHL PAI-1 during Sutureless Partial Nephrectomy in Mice Reduces Bleeding

**DOI:** 10.1155/2015/392862

**Published:** 2015-03-26

**Authors:** Khaled Shahrour, Rick Keck, Jerzy Jankun

**Affiliations:** ^1^Department of Urology, the University of Toledo, Health Science Campus, 3000 Arlington Avenue, Toledo, OH 43614, USA; ^2^Department of Clinical Nutrition, Medical University of Gdańsk, Debinki 7, 80-211 Gdańsk, Poland; ^3^Protein Research Chair, Department of Biochemistry, College of Sciences, King Saud University, Riyadh 11451, Saudi Arabia; ^4^Urology Research Center, Department of Urology, the University of Toledo, Health Science Campus, 3000 Arlington Avenue, Toledo, OH 43614, USA

## Abstract

PAI-1 prevents lysis of blood clot by inhibiting the urokinase and tPA induced conversion of plasminogen to plasmin. VLHL PAI-1 protein mutant was created to extend half-life over 700 hours. The objective of this paper was to test VLHL PAI-1 effects on bleeding during partial nephrectomy in mice. All animals had a left partial nephrectomy after intravenous infusion of saline or tPA. The animals were divided into four groups. Group 1 was infused with saline and kidney was exposed to saline too; Group 2 was infused with saline and kidney was exposed to PAI-1. Group 3 was infused with tPA and kidney was exposed to saline, while Group 4 was infused with tPA and kidney was exposed to PAI-1. Preweighed gauze containing PAI-1 or saline was then applied to the kidney for 30 minutes. The gauze was afterward weighed and blood loss was measured by subtracting the preweight of gauze from the final weight. We have observed a statistically significant (*P* ≤ 0.05) reduction of bleeding in PAI-1-treated group in comparison to saline and tPA-treated groups. Based on these results we propose that VLHL PAI-1 can be used therapeutically in limiting the flow of blood from renal wounds.

## 1. Introduction

Partial nephrectomy is the gold-standard surgical treatment of renal masses, especially those smaller than 4 cm [1]. The procedure entails cutting the mass out of the kidney and suturing the kidney back to close the kidney defect. Hemorrhage is a common complication that can occur intraoperatively or postoperatively due to the high blood flow that the kidney receives [2]. Intraoperative hemorrhage can occur during excision of deep tumors despite clamping the hilum. Immediate postoperative bleeding is related to a blood vessel that is incidentally incised during tumor resection and was not secured during kidney closure (probably missed due to vasospasm) and starts to bleed after the resolution of vasospasm. Delayed postoperative bleeding is mainly due to the formation of a pseudoaneurysm during the healing process of the kidney [3]. Improving intraoperative hemostasis should lower the hemorrhage rates related to partial nephrectomy. At the same time, if the surgeon is excising a tumor with minimal blood loss, the tumor margins will be better visualized and there will be less potential for positive margins. So improving intraoperative hemostasis during tumor excision should improve surgical outcome and decrease the chance and severity of complications. There are various agents that are available for hemostasis during partial nephrectomy but none of these agents have any prolonged effect in the postoperative period and they are usually not sufficient for hemostasis without closing the defect with suture placement.

Plasminogen activator inhibitor-1 (PAI-1) encoded by the SERPINE1 gene is also known as serpin E1 or endothelial plasminogen activator inhibitor. The major function of PAI-1 is inhibition of tissue plasminogen activator (tPA) and urokinase (uPA), activators of plasminogen to plasmin. PAI-1 is the principal inhibitor of the plasminogen activators, and the other plasminogen activator inhibitor-2 (PAI-2) is secreted by the placenta and is only present in substantial amounts during pregnancy [4–6]. Protease nexin-1 (PN-1) regulates matrix accumulation and coagulation under pathophysiologic conditions by inhibiting thrombin, plasmin, tPA, and uPA [7–9]. The major physiological function of plasminogen system (PAI-1, tPA, plasminogen, and others) is to regulate physiological fibrinolysis. PAI-1 prevents premature lysis of blood clots by tPA activated plasmin [10, 11].

Hemostasis depends on a delicate balance of coagulation and fibrinolysis factors. Dysfunction in either one can lead to excessive bleeding or clotting. For example, patients with defective PAI-1 protein or with PAI-1 deficiency bleed excessively. In these patients normal primary hemostasis is observed and a normal thrombus is formed but it is quickly lysed as there is no inhibitor to moderate tPA plasmin activation [12–14]. Also, elevated PAI-1 levels lead to hypofibrinolysis and in extreme cases to the development of arterial thrombotic events as an insufficient amount of plasmin is formed [15–17]. Intravenous bolus injections of wild-type PAI-1 (wPAI-1) markedly inhibit fibrinolysis in a dose responsive manner in vivo and stabilize a developing thrombus [10, 18–21]. One obstacle in using wPAI-1 as a hemostatic drug is its very short half-life (*t*_1/2_ = 2 h). Several PAI-1 mutants were created to extend half-life up to over 700 h with full inhibitory activity [22, 23]. Long time active PAI-1 mutants can be used therapeutically in limiting/stopping the flow of blood from wounds. The objective of the study is to evaluate the effect of PAI-1 on hemostasis during partial nephrectomy in mice.

## 2. Materials and Methods

### 2.1. Proteins

Fully active human tPA, product number HTPA-TC, was purchased from Molecular Innovations, Novi, MI. Very long half-life plasminogen activator type one (VLHL PAI-1) was produced in our laboratory. The mutation of two amino acids (Gln197→Cys, Gly355→Cys) in human wPAI-1 (SwissPROT P05121 [24]) produces fully active VLHL PAI-1 for over 700 h [22, 25]. Construction of this protein and purification was previously described by our group [10, 26]. Briefly, a bacmid containing VLHL PAI-1 DNA was used to transfect Sf9 cells [27], cells were harvested and lysed by two freeze-thaw cycles, and the lysate was centrifuged at 3000 ×g for 20 min to remove cellular debris. The supernatant was loaded onto a nickel resin packed column (GradiFrac System, Pharmacia Biotech) and the column was washed with buffer containing 40 mM imidazole in native buffer (50 mM NaH_2_PO_4_, 0.5 M NaCl, pH 8.00, containing protease inhibitors) until A_280_ reached the baseline. The protein was then eluted using a gradient of 40–250 mM imidazole in native buffer [28, 29]. The peak fractions were concentrated and further purified on HPLC (Millipore) Superose 12 FPLC column by elution with native buffer and concentrated to 2.5 mg/mL. Fully functional VLHL PAI-1 was stored at −80°C till used [18].

### 2.2. VLHL PAI-1/tPA Complex Formation

It was confirmed by SDS-PAGE electrophoresis at room temperature using 4–12% SDS-polyacrylamide gradient gels under nonreducing conditions. Gels were stained with Colloidal Coomassie Blue (Invitrogen, Grand Island, NY, USA).

### 2.3. Analysis of Clot Formation with Thromboelastography (TEG)

Blood was purchased from Bioreclamation Inc., Westbury, NY, and shipped cold. Sodium citrated mouse (C57BL/6J, cat. MSEWBCIT) whole blood was collected on day 0 from 3 different animals. Activity of tPA and VLHL PAI-1 in blood was measured by thrombelastography. This method not only allows for the measurement of global coagulation profile but also yields data on the kinetics and dynamics of clot formation and clot lysis in whole blood [30, 31]. The critical part of this instrument is a pin hanging on a torsion wire and suspended in a cup holding a blood sample (380 *μ*L). This pin oscillates at 6 rpm at a 4°45′ angle at 37°C and while blood changes viscosity in the course of clot formation the pin motion is progressively restrained [32]. In TEG assay 1 mL of blood was mixed with 20 *μ*L of kaolin (Haemoscope Co., Niles, IL). Next, 360 *μ*L of the mixture was transferred to a TEG cup containing 20 *μ*L of CaCl_2_ (0.2 M) and 20 *μ*L of either saline (0.15 M), tPA (0.5 *μ*g/mL), or tPA with VLHL PAI-1 (1.4 *μ*g/mL) [33, 34]. Under such controlled conditions lysis is measured and it is related to the bleeding time [10, 31, 35].

### 2.4. Animals

All experiments using animals in this study were approved by the University of Toledo Institutional Animal Care and Use Committee and done under the guidance of the Department of Laboratory Animal Resources, which is accredited by the Association for Assessment and Accreditation of Laboratory Animal Care (AAALAC) International. Animals used were 11-week-old, C576BL/6J male mice (Jackson Laboratory, Bar Harbor, Maine). A presurgical assessment was performed, consisting of a general observation to assess normal weight, body conformation, and behavior. Food and water were available* ad libitum*.

### 2.5. Hemostatic Agent

Small sections of gauze (50 × 20 mm) were preweighed and saturated with 0.25 mL of VLHL PAI-1 (300 *μ*g/mL) or saline immediately prior to clamping and resection of the kidney.

### 2.6. Systemic Administration

**Figure 1:**
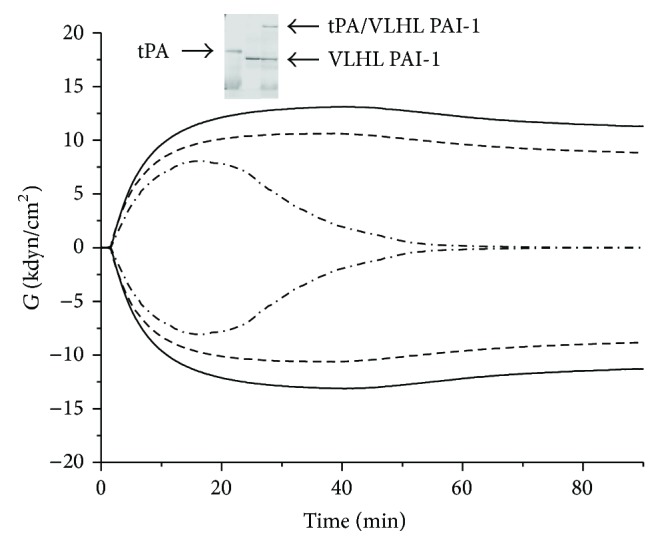
Plasma, solid line (no tPA, no VLHL PAI-1), clotted blood with mouse tPA alone (tPA 0.5 *μ*g/mL), dash dot line, and clotted blood with mouse tPA (tPA 0.5 *μ*g/mL) + VLHL PAI-1 (VLHL PAI-1  1.4 *μ*g/mL), short dash line. Insert shows complex formation of VLHL PAI-1 with tPA. Lane 1, mouse tPA; lane 2, VLHL PAI-1; lane 3, mouse tPA + VLHL PAI-1.

Animals were anesthetized via gas inhalation (1.0–3.0% isoflurane). The surgical area was shaved. The right or left jugular vein was surgically exposed and cannulated with a PE 10 catheter to administer tPA (tissue plasminogen activator) or saline (Figure 1). The catheter was connected to a syringe pump (Medex, Duluth, GA) to deliver tPA (30 *μ*g) or saline at 1.2 mL/hr. All animals received the same volume of fluids.

### 2.7. Partial Kidney Resection and Application of Hemostatic Reagent

**Figure 2:**
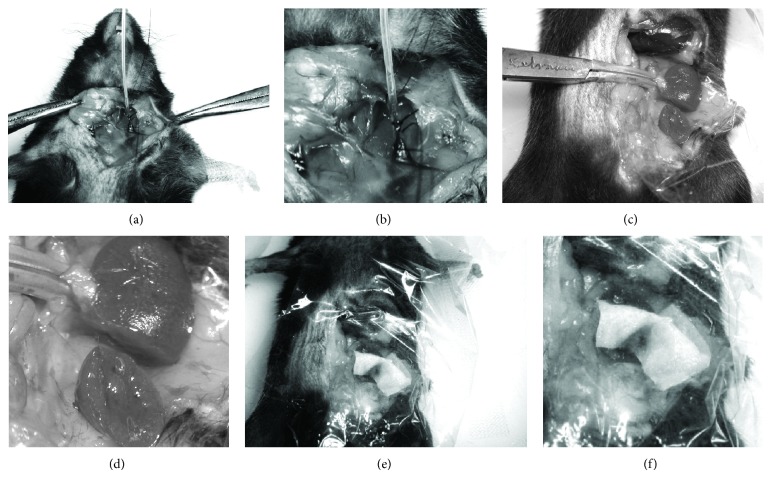
((a) and (b)) Cannulation of jugular vein in preparation for infusion of tPA or saline. ((c) and (d)) Clamping of renal vascular pedicle and partial nephrectomy. ((e) and (f)) Placement of PAI-1 or saline saturated gauze onto the surface of the renal injury.

Fifteen minutes after starting the tPA infusion, a midline incision was made to expose the left kidney. The kidney was freed from surrounding tissue. Clear plastic wrap was placed under the isolated kidney to contain any blood loss. An atraumatic clamp was placed across the renal vascular pedicle while a partial nephrectomy of the lower one-third of the kidney was performed as described in the literature, using a standardized heminephrectomy model where the kidney is cut transversely, visually aiming for the lower one-third of the parenchyma [36–38]. Gauze containing the VLHL PAI-1 or saline was applied to the injury site during clamping time of 30 seconds. The plastic wrap was pulled up over the kidney to prevent any moisture evaporation (Figure 2). After 30 minutes, the renal vessels were clipped and the plastic wrap containing the resected kidney and any blood was immediately removed and the animal was euthanized. The gauze was removed from the kidney and weighed and any excess blood not absorbed by the first gauze was soaked up with another preweighed piece of gauze (Figure 1). Blood loss was measured by subtracting the preweight of all gauze pieces from the final weight. The weight of the resected piece of kidney and remaining kidney was recorded for later analysis of variability in the resection.

### 2.8. Treatment and Control Groups

Four groups of animals were used as shown in Table 1. Group 1 was infused with saline for 30 minutes while injured kidney was exposed to saline (*n* = 3). Group 2 was infused with saline for 30 minutes, while injured kidney was exposed to VLHL PAI (*n* = 3). Group 3 was infused with tPA (30 *μ*g) for 30 minutes, while injured kidney was exposed to saline (*n* = 8). Group 4 was infused with tPA (30 *μ*g) for 30 minutes, while injured kidney was exposed to VLHL PAI-1 (*n* = 10).

### 2.9. Statistical Analysis

It was carried out using Origin 8 program (OriginLab Corporation, Northampton, MA 01060). Statistical significance was set at a *P* < 0.05 as calculated using ANOVA test.

## 3. Results and Discussion

Activity of VLHL PAI-1 was confirmed by two independent methods. Ability of complex formation between human VLHL PAI-1 and mouse tPA was corroborated by SDS PAGE electrophoresis. The characteristic band in molecular weight equal to sum of tPA and VLHL molecular weights was seen on the stained gel (Figure 1, insert). Also, as seen in the clotting profile (Figure 1), the addition of tPA to clotted blood resulted in clot lysis, but the addition of tPA and VLHL PAI-1 prevented blood clot lysis.

**Figure 3:**
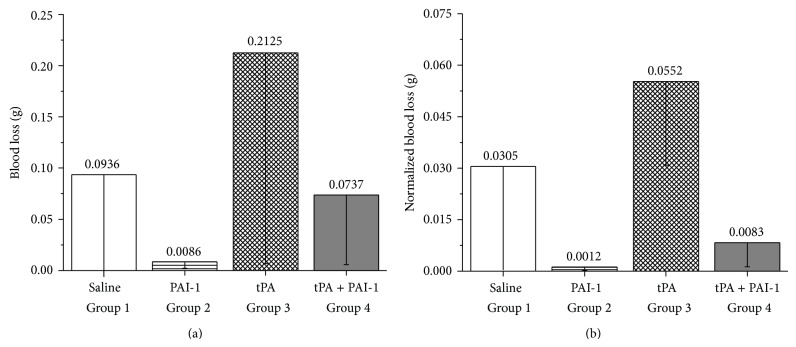
(a) Total blood loss; (b) normalized total blood loss. Saline treated: Group 1, VLHL PAI-1 treated: Group 2, mouse tPA treated: Group 3, and mouse tPA + VLHL treated: Group 4. Blood loss was normalized by adjusting volume of blood by ratio of weight of the resected piece of kidney to remaining kidney. Statistical significance of differences between different treatment groups are in Table 2.

Stanching of blood flow from wounds after PAI-1 application has been reported by Racanelli et al. [39] as well as by us in the past [18] on different animal models. However, these studies deal with much lesser wounds than caused when extensive kidney resection is done. A major drawback to some of the earlier studies was the use of wild-type PAI-1 which converts into the latent, inactive form in *t*_1/2_ ~ 2 h [39]. The therapeutic applications become possible only when PAI-1 mutants with longer half-life were developed [22, 40]. Bleeding in control animals (Group 1) as can be seen in Table 1 and Figure 3 was excessive. We used VLHL PAI-1, which remains fully active for more than 700 h that statistically reduced bleeding (Group 2) when compared to control groups. Only three animals in each group were sufficient to establish a statistically significant reduction of bleeding in PAI-1 treated animals. Group 3 was a positive control where tPA was systemically applied to induce excessive fibrinolysis resulting in increased bleeding. In our study, adding tPA mimicked clinical situations where it has been reported that tPA and/or uPA plasminogen activators are overexpressed on mRNA or protein level in renal cancer or after surgery [41–43]. As expected tPA extensively increases bleeding from partially nephrectomized kidneys as compared with control and PAI-1 treated groups.

There are large numbers of therapeutics that induce blood clots and reduce bleeding [24, 44, 45]. Much less abundant are antifibrinolytic agents that inhibit plasmin responsible for clot lysis. These include *ε*-aminocaproic acid, tranexamic acid, and aprotinin, which has limited availability due to its side effects profile [44–47]. Most small molecular inhibitors are not specifically acting on different proteases and are not always effective [39, 48, 49]. As opposed to the small molecule inhibitors, PAI-1 is specific for PAs and acts by making a 1 : 1 complex, followed by the formation of a covalent bond between the active site of the protease and the reactive center of the serpin. Furthermore, PAI-1 binds to the fibrin but not to fibrinogen and thus can localize itself at sites of injury [49]. Also, PAI-1 retains its complete tissue-type plasminogen inhibitory activity protecting the clot from premature dissolution at sites of fibrin deposition [50]. Therefore, VLHL PAI-1 may be of value in reducing blood loss including situations where excessive fibrinolysis contributes to bleeding or tPA induced bleeding during thrombolytic therapy. No differences between topical and systemic applications of PAI-1 and aprotinin have been reported in the literature [51, 52]. The topical application of proteins should be preferred due to the promise of fewer side effects than systemic delivery.

One critique to the current study would be that PAI-1 effects in mice may not be replicated in human or other large animals as previously demonstrated that knockout PAI-1 mice did not demonstrate the same findings as patients with PAI-1 deficiency [53]. Hence, further experimentation with larger animals, such as pigs, should be done based on the findings of this study to open the possibility of clinical applications of VLHL PAI-1. Other potential urologic applications of PAI-1 that can be studied in the future include intractable hematuria and percutaneous nephrolithotomy.

## Figures and Tables

**Table 1:** Experimental groups.

Group	Infusion	Nephrectomy exposure	*n*
1	Saline	Saline	3
2	Saline	PAI-1	3
3	tPA	Saline	8
4	tPA	PAI-1	10

**Table 2:** Statistical significance of differences between different treatment groups.

	*P* value for blood loss	*P* value for normalized blood loss
Group 1 (saline/saline) versus Group 2 (saline/PAI-1)	0.041	0.039
Group 1 (saline/saline) versus Group 3 (tPA/saline)	0.005	0.002
Group 3 (tPA/saline) versus Group 2 (saline/PAI-1)	NS	0.009
Group 3 (tPA/saline) versus Group 4 (tPA/PAI-1)	NS (0.051)	0.017

Blood loss was normalized by adjusting volume of blood by ratio of weight of the resected piece of kidney to remaining kidney.
